# Physical activity, resilience, and depressive symptoms in Chinese college students: gender differences in mediating pathways'

**DOI:** 10.3389/fpsyg.2025.1726761

**Published:** 2026-01-26

**Authors:** Junshuai Xu, Yi Yang, Xiudong Wei

**Affiliations:** 1 Graduate School, Jose Rizal University, Mandaluyong, Philippines; 2 College of Physical Education, Southwest University, Chongqing, China; 3 College of Physical Education, Chongqing University of Arts and Sciences, Chongqing, China

**Keywords:** college students, depression, gender difference, physical activity, resilience

## Abstract

**Objective:**

This study aims to explore the relationship between physical activity and depression levels among college students, while examining the mediating role of resilience and the impact of gender differences.

**Methods:**

Using measurement tools such as the Physical Activity Rating Scale (PARS-3), the Self-Rating Depression Scale (SDS), and the Adolescent Resilience Scale, a questionnaire survey was conducted among 2,000 college students in Chongqing, China. The data were processed and analyzed using SPSS 26.0, including difference analysis, correlation analysis, regression analysis, and Bootstrap analysis, and the structural equation model was established using AMOS 26.0 software.

**Results:**

(1) From the perspective of gender differences, male college students exhibit a significantly higher level of physical activity than female students, while also demonstrating significantly lower levels of depression. (2) There is a significant correlation between physical activity, resilience, and depression levels of college students. (3) The level of physical activity exhibits a direct negative correlation with depression levels among college students, with resilience playing a partial mediating role between the two. (4) In the mediation models stratified by gender, physical activity demonstrates greater direct explanatory power for depression among male students, whereas female students exhibit a stronger mediated explanatory power.

**Conclusion:**

Regular participation in physical activities is an important way to improve the mental health level of college students. As much as possible, college students should be encouraged and supported to participate in physical activities (such as organizing fun sports meetings, sports clubs), which holds positive direct or indirect implications for preventing or reducing the incidence of depression.

## 1 Introduction

In recent years, the incidence of depression has been increasing year by year, and the World Health Organization regards depression as one of the important factors for premature death and disability worldwide (Shorey et al., 2022). It is a common and serious mental illness, mainly characterized by persistent depression, loss of interest, and anhedonia, which can significantly affect an individual's daily life (Li et al., 2022). Among them, the incidence of depression among college students is higher than that in the general population and shows a rapid upward trend (Shi et al., 2024), and it is a very common emotional disorder among college students (Chaliawala et al., 2025). For example, the result of a meta-analysis involving 33,565 samples showed that the detection rate of depression among Chinese college students reached 24.7% (Wang et al., 2020). Meanwhile, the “Report on the Mental Health Development of Chinese Citizens (2021–2022)” shows that the detection rate of depression risk in the 18–24 age group is as high as 24.1%, among which college students are a high-risk group for depression (Fu et al., 2023). College students are in an important transitional stage between adolescence and adulthood, usually facing multiple pressures in terms of studies, employment, and interpersonal relationships. If not handled properly, it is very easy to have depressive emotions (Sun M. Y. et al., 2024; Yang X. F. et al., 2025). Studies have found that depression pose significant harm to individuals, such as exacerbating mental health problems, increasing the risk of suicide, affecting normal sleep, and interfering with social functions (Schulz, 2020; Ribeiro et al., 2018). For example, college students who have been in a state of depression for a long time will not only encounter problems such as reduced learning efficiency and difficulties in interpersonal communication but may even exhibit behaviors that endanger their lives, such as self-harm and suicide (Zhao et al., 2021). Further research indicates that females are more susceptible to depression than males (Kuehner, 2017), which this may be attributed to earlier physiological development in females and their heightened emotional sensitivity and vulnerability (Yang, 2023). Additionally, academic failure poses a higher risk of triggering depression in female students compared to males (Mccarty et al., 2008). Therefore, exploring effective ways to reduce the incidence of depression among college students and improve depression based on gender differences is an important measure to promote the healthy growth of college students.

Insufficient physical activity is a risk factor for depression among college students (Li et al., 2020). A study has shown that insufficient physical activity may lead to a decline in an individual's emotional regulation function by increasing stress hormones and disrupting the balance of neurotransmitters such as serotonin and dopamine, thereby causing emotional disorders such as depression and anxiety (Li et al., 2023). A follow-up study involving 2,134 Chinese college students showed that a lack of physical activity was associated with the occurrence of depression (Xie et al., 2019). Importantly, regular physical activity is believed to play an effective role in preventing and improving depression among college students. For example, physical activity can significantly reduce negative emotions such as depression and anxiety, and help regulate emotions and maintain mental health (Schuch et al., 2018). A cross-sectional study found that active participation in physical activities was negatively correlated with the risk of depression among college students (Yang J. W. et al., 2025; Xiang et al., 2020), and the evidence from cohort studies also has similar findings (Pereira et al., 2014; Kandola et al., 2020). Furthermore, there is a linear relationship between physical activity and depression levels among college students, and it is manifested that compared with low participation in physical activities, college students who engage in more physical activities have a risk of developing depression that can be reduced by more than 34.2% (Xu et al., 2024). However, some studies suggest that vigorous physical activity is significantly associated with reduced depression only in males, with no such correlation observed in females (Goldfield et al., 2011). Concurrently, (Xiao et al. 2017) found that increased physical activity levels helped alleviate depression in male students, whereas no significant difference in the prevalence of depression was observed across different activity levels among female students. The results indicate a linear relationship between physical activity and depression in college students, though this association may be subject to gender differences.

To more effectively reveal the relationship between physical activity and depression among college students, researchers have attempted to explore the underlying mechanisms between the two from both physiological and psychological perspectives. From a physiological perspective, studies have shown that physical activity can significantly affect the secretion of neurotransmitters in the brain, such as promoting the release of neurotransmitters like dopamine, serotonin, and endorphins, and these neurotransmitters play a key role in regulating emotions and stress responses (Schuch et al., 2016; Gao et al., 2025). Studies from imaging have shown that the volume of the hippocampus in the brains of patients with depression is smaller compared to that of normal people (Herzberg et al., 2024), and physical activity can increase the volume of some areas of the hippocampus in individuals (Hendrikse et al., 2022). From the psychological perspective, the research found that social adaptation (Wang et al., 2024), personality traits (Guo and Ma, 2022), the satisfaction of basic psychological needs (Jiang et al., 2025), group identity (Greenaway et al., 2015), and psychological resilience (Reivich et al., 2013) all have varying degrees of correlation with depression. Among them, as a positive psychological quality, the relationship between resilience and depression among college students has attracted much attention, and whether there are gender differences in this relationship has also become a key focus of research. Resilience is the ability or trait of an individual to cope with stress, setbacks, or trauma (Garcia-Dia et al., 2013), and is regarded as a protective factor that inhibits an individual from developing depression (Myntti and Armstrong, 2022). The protective factor model points out that in the process of interaction between individuals and risk environments, resilience can promote individuals to effectively regulate their cognition and emotions, enhance environmental adaptability, and may be one of the important moderating variables in the negative impact of risk factors on individuals (Marten, 2025). Interestingly, studies have found that physical activities have positive correlations of varying degrees with the resilience of different groups of people, including children (Granizo et al., 2019), patients with attention deficit hyperactivity disorder (Liang et al., 2023), and college students (Xu et al., 2021; Li and Guo, 2023). Further studies have shown that resilience has a mediating effect between physical activity and negative emotions among college students (Liu et al., 2024; Zuo et al., 2025), but it is currently not clear whether there are gender differences in this mediating role.

To sum up, physical activity seems to be an effective strategy for preventing or reducing depression among college students, but whether there are gender differences in the linear relationship between the two remains unclear at present. Meanwhile, the role that resilience, as a positive psychological quality for coping with stress, plays between physical activity and depression still needs to be further explored. Based on this, this study aims to investigate the relationship between physical activity and depression among college students, as well as gender differences, and to reveal the role of resilience therein. The hypotheses proposed in this study are as follows: (H1) There is a negative correlation between physical activity and depression among college students. (H2) There is a positive correlation between physical activity and the resilience of college students. (H3) Resilience plays a mediating role between physical activity and depression among college students. (H4) There are gender differences in the relationship between physical activity, resilience, and depression among college students.

## 2 Participants and methods

### 2.1 Participants

This study employed a cross-sectional survey design and conducted questionnaire surveys among full-time undergraduate students from three comprehensive universities in Chongqing, China. Stratified random sampling was performed based on academic majors, excluding students majoring in sports (to avoid significant differences in physical activity levels among the samples). Specifically, based on the total number of full-time undergraduate students at the three universities, proportional sampling was conducted for each major, followed by stratified random sampling of students from each grade level (freshman, sophomore, junior, and senior) according to their student ID numbers. To ensure the quality and reliability of the questionnaire filling, after a detailed explanation of the precautions and requirements for filling out the questionnaire to the students, the participants were required to complete the questionnaire independently within 10 min based on their actual situation and collect it on the spot. Furthermore, to enable participants to have sufficient time to fill out the questionnaires, the distribution and filling of the questionnaires were carried out in the classroom during the break time. A total of 2,000 questionnaires were distributed in this study, and 1,868 were retrieved, with a recovery rate of 93.40%. Through steps such as descriptive statistics, numerical transformation, and processing of missing values, 129 invalid samples with incomplete key information, random filling, and missing data were eliminated, and 1,739 valid questionnaires were obtained, with an effective rate of 93.09%. Among them, there were 952 male (54.74%), with an average age of 20.18 ± 2.02 years, an average height of 1.73 ± 0.09 m, and an average weight of 64.76 ± 14.58 kg. There were 787 female (45.26%), with an average age of 19.66 ± 1.85 years, an average height of 1.61 ± 0.87 m, and an average weight of 53.68 ± 12.76 kg. This study followed the Declaration of Helsinki, obtained written informed consent forms from all participants, and all participants participated voluntarily.

### 2.2 Measuring tools

#### 2.2.1 Physical activity rating scale (PARS-3)

Physical activity levels of participants were assessed using the PARS-3 developed by (Liang 1994). The scale is in Chinese and has been validated for use among Chinese university students (Wang et al., 2024). This scale has a total of 3 items, including physical activity intensity, frequency, and time. The Likert five-point scoring system was adopted, with both intensity and frequency divided into 1 to 5 score levels, and time divided into 0 to 4 score levels. The total physical activity score of the participants was quantified by the formula “physical activity intensity × time × frequency”, and the score range was 0 to 100 points; the higher the score, the greater the physical activity. In this study, the test-retest reliability *r* of this scale was 0.82, the factor load was greater than 0.5, the AVE was greater than 0.5, and the combined reliability CR was greater than 0.6. Meanwhile, the Cronbach's α coefficient was 0.81, indicating that this scale has good reliability and validity.

#### 2.2.2 Self-rating depression scale (SDS)

The depression levels of the participants were evaluated using the SDS developed by (Zung 1965) and verified by (Campo-Arias et al. 2022). The scale has been translated, applied, and validated for use among Chinese university students (Zhang et al., 2024). This scale has a total of 20 items, and it is a one-dimensional scale. The Likert four-point scoring system was adopted, with scores ranging from “no or very little time” to “most or all time” ranging from 1 to 4, respectively. The standard total score of the scale was calculated by adding up the scores of 20 items and then multiplying by 1.25 (the scores were rounded to the nearest whole number), and the higher the score, the more severe the depression of college students. A standard total score of less than 53 indicates no depression, 53–62 indicates mild depression, 63–72 indicates moderate depression, and ≥73 points indicates severe depression. In this study, the test-retest reliability of this scale was *r* = 0.87, the factor load was greater than 0.5, the AVE was greater than 0.6, and the combined reliability CR was greater than 0.6. Meanwhile, the Cronbach's α coefficient of this scale is 0.85, indicating that this scale has good measurement reliability and validity.

#### 2.2.3 Adolescent resilience scale (ARS)

The resilience of participants was assessed using the ARS compiled by (Hu and Gan 2008). The scale is in Chinese and has been validated for use among Chinese adolescents (Fan et al., 2025). This scale consists of 27 items, and it contains a total of five dimensions, namely goal focus (five items), emotion control (six items), positive cognition (four items), family support (six items), and interpersonal assistance (six items). This scale was quantified using the Likert five-point scale. According to the options “completely inconsistent to completely consistent”, they were scored from 1 to 5 points (among them, 12 items are scored in reverse), respectively, with the score range being 27–135 points. The higher the score, the better the resilience. In this study, the test-retest reliability was *r* = 0.85, the loads of each factor were all greater than 0.5, the AVE was greater than 0.7, and the combined reliability CR was greater than 0.6. Meanwhile, the Cronbach's α coefficient of the total scale was 0.83, among which the Cronbach's α coefficients of the five dimensions of goal concentration, emotion control, positive cognition, family support, and interpersonal assistance were 0.81, 0.83, 0.87, 0.79, and 0.80, respectively. The results of confirmatory factor analysis were as follows: χ^2^/*df* = 2.13, RMSEA = 0.05, AGFI = 0.94, TLI = 0.97, CFI = 0.98, IFI = 0.96, GFI = 0.97. It indicates that this scale has good measurement reliability and validity.

### 2.3 Statistical methods

In this study, SPSS 26.0 was used to process and analyze the data, including difference tests, and reliability and validity tests. Meanwhile, Pearson correlation analysis, regression analysis, and Bootstrap analysis were used to investigate the relationships among the variables, and AMOS 21.0 was employed to establish a structural equation model to examine the mediating role of resilience between physical activity and depression. The significance levels of all indicators were set at *p* < 0.05.

## 3 Results

### 3.1 Common method deviation test

This study adopted the questionnaire survey method, to minimize the influence of common method bias on the results as much as possible, during the testing, this study adopted methods such as anonymous questionnaire measurement, forward and reverse scoring, and standardized testing for the corresponding control. After the data collection was completed, this study also adopted the Harman single-factor test method to investigate the common method bias problem (Podsakoff et al., 2003). The results show that after factor analysis of all variables, there are 7 factors with characteristic roots greater than 1, and the variation explained by the first factor is 31.08%, which is less than the critical criterion of 40%. Therefore, there is no common method bias problem for verification in this study.

### 3.2 Analysis of the characteristics of college students' physical activity, resilience, and depression

In Table 1, (1) In terms of gender comparisons, significant differences were observed in both physical activity (*t* = 6.60, *p* < 0.05, Cohen's *d* = 0.32) and depression levels (*t* = 8.30, *p* < 0.05, Cohen's *d* = 0.40) among college students. Specifically, male students manifest significantly higher levels of physical activity and significantly lower levels of depression compared to female students. However, there was no significant difference in resilience (*t* = 2.91, *p* > 0.05, Cohen's *d* = 0.14). (2) In terms of grade comparison, there were no significant grade differences among college students in physical activity (*F* = 1.97, *p* > 0.05, *Partial* η^2^ = 0.01), depression (*F* = 2.08, *p* > 0.05, *Partial* η^2^ = 0.02), and resilience level (*F* = 1.52, *p* > 0.05, *Partial* η^2^ = 0.01).

**Table 1:** Demographic differences in physical activity, resilience, and depression.

**Category**	**Variable**	**Physical activity**	**Resilience**	**Depression**
Gender	Male	30.13 ± 5.22	62.26 ± 8.13	54.39 ± 7.33
	Female	27.56 ± 7.91	61.15 ± 8.01	57.47 ± 8.20
	*T*	6.60^*^	2.91	8.30^*^
	*Cohen's d*	0.32	0.14	0.40
Grade	Freshman	29.06 ± 5.18	61.33 ± 7.67	55.69 ± 7.52
	Sophomore	28.92 ± 4.35	60.02 ± 7.18	54.78 ± 6.69
	Junior	27.63 ± 4.11	59.68 ± 7.55	56.47 ± 7.33
	Senior	28.20 ± 4.86	61.89 ± 7.92	57.10 ± 7.94
	*F*	1.97	1.52	2.08
	*Partial η^2^*	0.01	0.01	0.02

^*^ *p* < 0.05.

### 3.3 Correlation analysis of college students' physical activity, resilience, and depression

Correlation analysis showed (Table 2) that the level of physical activity of college students was significantly positively correlated with resilience (*r* = 0.36, *p* < 0.01), and significantly negatively correlated with depression (*r* = −0.48, *p* < 0.001). Meanwhile, resilience was significantly negatively correlated with the level of depression (*r* = −0.32, *p* < 0.01). The correlations among the main variables all reached a significant level, which provided a good basis for the subsequent test of the mediating role.

**Table 2:** Correlation coefficients of physical activity, resilience, and depression.

**Variable**	***M* ±*SD***	**Physical activity**	**Resilience**	**Depression**
Physical activity	28.85 ± 8.03	1	1	1
Resilience	61.70 ± 8.07	0.36^**^		
Depression	55.93 ± 7.76	−0.48^***^	−0.32^**^	

^**^ *p* < 0.01, and ^***^ *p* < 0.001.

### 3.4 Analysis of the relationship between physical activities and depression and its mediating role

#### 3.4.1 Direct role analysis

General linear regression analysis was used to test the direct relationship among variables (Table 3). Firstly, after controlling for demographic variables such as gender and grade in this study, the direct relationship of physical activities on the resilience and depression of college students were examined, respectively. The results revealed that there is a direct positive relationship between physical activity and resilience (β = 0.36, *p* < 0.01), which could explain 12% of the variation. Moreover, a direct negative relationship exists between physical activity and depression (β = −0.48, *p* < 0.001), which can explain 21% of the variation. Finally, taking resilience as the independent variable and depression as the dependent variable, it was found a direct negative relationship between resilience and depression (β = −0.32, *p* < 0.01), which could explain 9% of the variation.

**Table 3:** Linear regression analysis of physical activity and resilience on depression.

**Variable**	**Resilience**				**Depression**			
β	*R* ^2^	*Δ**R*** ^2^	**95% CI**	β	*R* ^2^	*Δ**R*** ^2^	**95% CI**
Physical activity	0.36^**^	0.13	0.12	(0.34, 0.39)	−0.48^***^	0.23	0.21	(−0.51, −0.46)
Resilience	–	–	–	–	−0.32^**^	0.10	0.09	(−0.35, −0.30)

^**^ *p* < 0.01, and ^***^ *p* < 0.001.

#### 3.4.2 The mediating role of resilience between physical activity and depression

**Figure 1:**
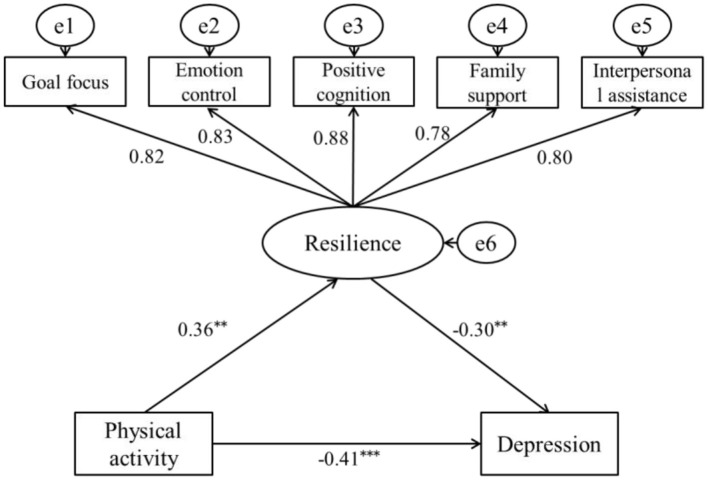
The mediating model diagram of resilience between physical activity and depression. ** *p* < 0.01, and *** *p* < 0.001.

The mediating role test method of (Baron and Kenny 1986) was adopted to investigate the mediating relationship. A structural equation model was established using AMOS software to investigate the mediating role of resilience between physical activity and depression among college students (Figure 1). The fitting indicators of the model are as follows: χ^2^/*df* = 2.02, RMSEA = 0.04, GFI = 0.95, TLI = 0.96, NFI = 0.96, IFI = 0.97, and AGFI = 0.98, indicating that the model has a good fit and is suitable for the mediating role test. The findings of this study indicate that, after incorporating resilience as a mediating variable, a direct positive relationship exists between physical activity and resilience (β = 0.36, SE = 0.04, *p* < 0.01), while a direct negative correlation is observed between resilience and depression (β = −0.30, SE = 0.03, *p* < 0.01). However, the path coefficient of physical activity on depression levels decreased but still reached a significant level (β = −0.41, SE = 0.02, *p* < 0.01). It indicates that resilience plays a partial mediating role between physical activity and depression levels among college students, and the decomposition of the value of each path is shown in Table 4 (Bootstrap was drawn from 5,000 samples). Therefore, the hypotheses H1, H2, and H3 of this study have all been confirmed.

**Table 4:** Decomposition of the path relationship of physical activity and depression.

**Relationship**	**Path**	**Standardized value**	**Proportion of the total value**	**Boot 95% CI**
Total value	–	−0.52	100%	(−0.55, −0.48)
Direct relationship	Physical activity → Depression	−0.41	78.85%	(−0.43, −0.39)
Indirect relationship	Physical activity → Resilience → Depression	0.36 × (−0.30) = −0.11	21.15%	(−0.14, −0.09)

#### 3.4.3 Gender differences in the mediating relationship model

This study revealed significant gender differences in physical activity and depression levels among college students. To further investigate whether such gender disparities also exist in the mediation model, separate mediation models were established for male and female students.

**Figure 2:**
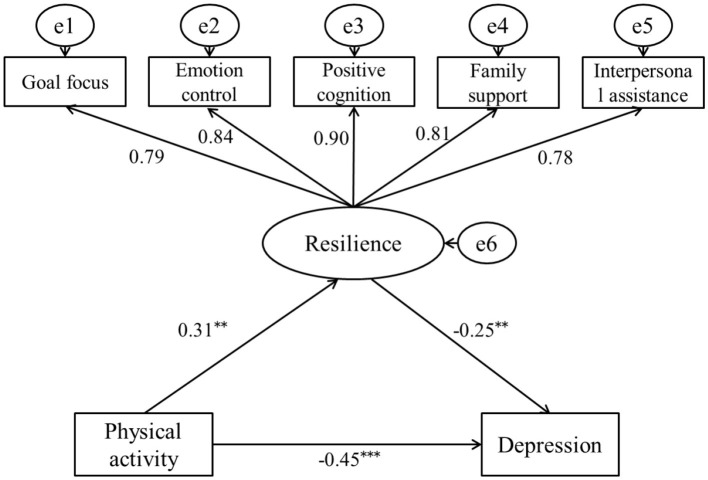
The mediating model of resilience between physical activity and depression in male college students. ** *p* < 0.01, and *** *p* < 0.001.

In the male student model, the fit indices were as follows: χ^2^/*df* = 1.98, RMSEA = 0.03, GFI = 0.97, TLI = 0.97, NFI = 0.99, IFI = 0.95, and AGFI = 0.98, indicating good model fit. The path coefficients indicate a direct negative relationship between physical activity and depression levels among male college students (β1 = −0.49, SE = 0.02, *p* < 0.001). After introducing resilience as a mediating variable, a direct positive relationship exists between physical activity and resilience (β = 0.31, SE = 0.03, *p* < 0.01), while a direct negative relationship is observed between resilience and depression (β = −0.25, SE = 0.03, *p* < 0.01). However, the path coefficient of physical activity on depression level decreased, but still reached a significant level (β2 = −0.45, SE = 0.02, *p* < 0.01), indicating that resilience plays a partial mediating role between physical activity and depression level in male college students. The decomposition of the value of each path is shown in Table 5, Figure 2.

**Table 5:** Decomposition of the path relationship of physical activity and depression in male college students.

**Relationship**	**Path**	**Standardized value**	**Proportion of the total value**	**Boot 95% CI**
Total value	–	−0.53	100%	(−0.56, −0.50)
Direct relationship	Physical activity → Depression	−0.45	84.91%	(−0.47, −0.42)
Indirect relationship	Physical activity → Resilience → Depression	0.31 × (−0.25) = −0.08	15.09%	(−0.11, −0.06)

**Figure 3:**
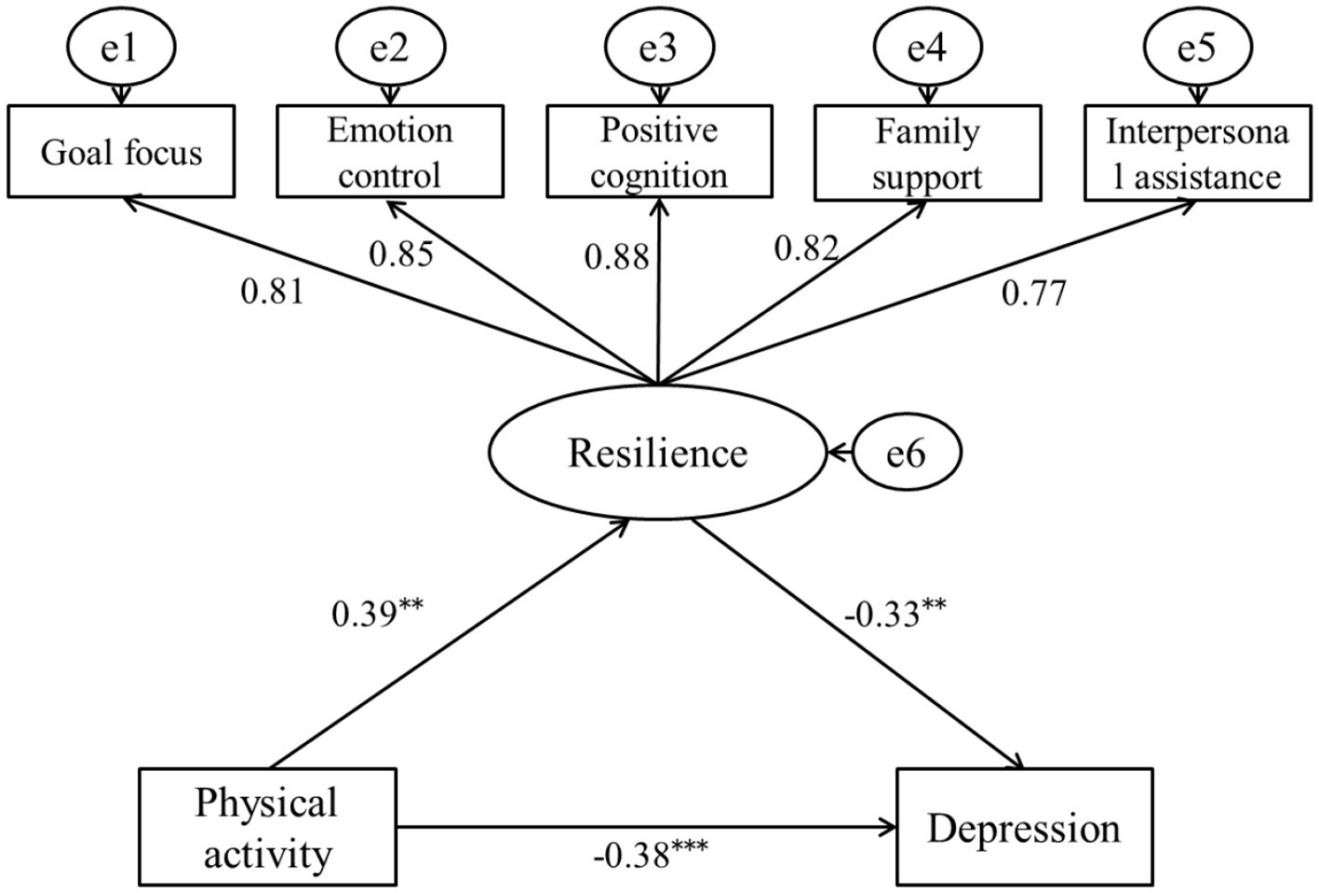
The mediating model of resilience between physical activity and depression in female college students. ** *p* < 0.01, and *** *p* < 0.001.

In the female student model, the fit indices were as follows: χ^2^/*df* = 2.08, RMSEA = 0.04, GFI = 0.96, TLI = 0.97, NFI = 0.98, IFI = 0.96, and AGFI = 0.97, indicating good model fit. The path coefficients indicate a direct negative relationship between physical activity and depression among male college students (β1 = −0.43, SE = 0.04, *p* < 0.001). After introducing resilience as a mediating variable, a direct positive relationship exists between physical activity and resilience (β = 0.39, SE = 0.02, *p* < 0.01), while a direct negative relationship is observed between resilience and depression (β = −0.33, SE = 0.02, *p* < 0.01). However, the path coefficient of physical activity on depression level decreased, but still reached a significant level (β2 = −0.38, SE = 0.03, *p* < 0.01), indicating that resilience still plays a partial mediating role between physical activity and the depression level in female college students. The decomposition of the value of each path is shown in Table 6. Therefore, Hypothesis H4 of this study has been confirmed (Figure 3).

**Table 6:** Decomposition of the path relationship of physical activity and depression in female college students.

**Relationship**	**Path**	**Standardized value**	**Proportion of the total value**	**Boot 95% CI**
Total value	–	−0.51	100%	(−0.54, −0.47)
Direct relationship	Physical activity → Depression	−0.38	74.51%	(−0.40, −0.35)
Indirect relationship	Physical activity → Resilience → Depression	0.39 × (−0.33) = −0.13	25.49%	(−0.16, −0.12)

## 4 Discussion

### 4.1 Analysis of the differences between physical activity and depression levels among college students

This study found, through comparison, that there were significant gender differences in physical activity and depression levels among college students, while there were no significant gender differences in resilience. On one hand, male students manifest significantly higher levels of physical activity and significantly lower levels of depression compared to female students. Research indicates that male college students outperform their female counterparts in terms of physical activity intensity, duration, and frequency, resulting in greater overall physical activity volume (Xiao et al., 2017). This finding aligns with gender-role stereotypes and behavioral patterns characterized by “male propensity for activity and female preference for tranquility” (Zhang and Dong, 2017). Furthermore, an examination of the content and form of physical activities chosen by college students reveals distinct gender-based patterns: male students tend to engage in active, competitive moderate-to-vigorous intensity activities, often committing to longer durations and higher frequencies (such as basketball and soccer matches). In contrast, female students predominantly select low-to-moderate intensity recreational activities (such as jogging, walking, and household chores) with an emphasis on leisure (Dong et al., 2013). On the other hand, this study found that male college students exhibited significantly lower levels of depression compared to females, which is consistent with previous research (Kuehner, 2017; Mccarty et al., 2008). A study indicates that gender differences in depression emerge during adolescence, with females experiencing approximately twice the prevalence rate of males (Yang et al., 2017). The researcher suggests that earlier physiological development in females, coupled with heightened emotional sensitivity and vulnerability, leads to greater internalization of emotions and consequently increased susceptibility to depression (Yang, 2023). Meanwhile, from the perspective of physiological mechanisms, the differences in brain structure and function (such as the frontal-limbic system and anterior cingulate gyrus) and the HPA axis between male and female students may mediate this gender difference (Wang and Qiu, 2023). However, this study did not find significant grade-level differences in physical activity, resilience, and depression levels among college students, indicating that grade is not the main factor affecting the physical activity and mental health of college students. As far as we know, on university campuses, college students of different grades can enjoy sports facility resources and freely arrange their physical activity time, so it may not directly affect their physical activity levels. Similarly, the academic pressure in university is relatively lower compared to high school, and there won't be much difference in academic tasks among different grades. Therefore, there are no significant grade-level differences in mental health levels, such as resilience and depression.

### 4.2 The direct relationship between physical activity and depression among college students

This study found that after controlling for demographic variables such as gender and grade level, a direct negative relationship exists between physical activity and depression among college students. This is relatively consistent with previous studies, such as two large-sample studies that have shown that participation in physical activities is negatively correlated with the incidence of depression, and those who actively participate in physical activities have a lower probability of developing depression (Jiazhi et al., 2025; Taliaferro et al., 2009). (Zhou et al. 2024) found that college students engaging in physical activity almost 3–4 times a day or a week help reduce the risk of anxiety and depression symptoms, indicating that a higher frequency of physical activity can effectively regulate physiological and psychological states, thereby alleviating emotional distress. Other studies have also reached similar conclusions, namely that moderate to high levels of physical activity help reduce the risk of depression (Li et al., 2024), and high levels of physical activity can reduce the risk of depressive and anxiety symptoms by 12% to 34% (Wolf et al., 2021). That is to say, there is a linear relationship between the physical activity of college students and the risk of depression, which is manifested as the higher the level of physical activity, the lower the risk of developing depression. Therefore, when college students develop depressive tendencies due to factors such as interpersonal relationships, academic pressure, emotional disorders, or internet addiction, maintaining regular moderate to high-intensity physical activities may be an effective measure to reduce the risk of depression.

### 4.3 The mediating role of resilience between physical activity and depression among college students

The results of this study show that physical activity not only affects the depression level of college students through a direct effect but also has an indirect impact on the depression level through part of the mediating effect of resilience. Resilience refers to an individual's effective coping and good adaptability when facing adversity, trauma, or other major life pressures (Lu et al., 2020). When facing various risk pressures such as family relationships and academic pressure, individuals with high resilience have fewer negative thoughts, such as suicidal ideation, and will actively transform risk pressures into the driving force for growth (Yang and Deng, 2015). Therefore, improving an individual's resilience may be an effective way to reduce the occurrence of their negative psychology and dangerous behaviors. On the one hand, research shows that physical activity has a positive correlation with the resilience of college students and can promote the improvement of resilience levels (Liu, 2020; Li et al., 2021). The resilience score of college students increases with the increase in physical activity, and the level of physical activity has a high correlation with the two dimensions of emotional control and goal concentration in the resilience of adolescents (Zhao et al., 2024). Among them, compared with participating in low-intensity physical activities, college students who participate in moderate and high-intensity physical activities usually have higher resilience (Song et al., 2014). This might be because physical activity can have a positive impact on brain structure and function, achieving better top-down self-regulation and thereby promoting the development of resilience in adolescents (Belcher et al., 2021). On the other hand, compared with adolescents with lower resilience, those with higher resilience have significantly lower levels of depression (Reivich et al., 2013; Niu et al., 2015). The theory of resilience holds that negative events do not necessarily trigger the emergence of negative emotions, and the key lies in whether an individual possesses protective resources for positive coping and handling (Dong, 2015). However, once the “defense line” of resilience is broken through, it is very likely to cause psychological adaptation problems in individuals, such as depressive tendencies (Zhang and Wang, 2022). This is in line with the view of the protective factor model (Marten, 2025). Among college students, resilience is considered to be significantly negatively correlated with depression, and depression in men are significantly higher than those in women (Jiang et al., 2023). A study on early adulthood shows that resilience is negatively correlated with depression in both healthy and depressed individuals, and resilience can negatively predict depression (Ye et al., 2018). Further research indicates that resilience has a partial mediating effect between physical activity and the negative emotional states of college students, and can partially explain the positive impact of physical activity on depression, anxiety, and stress (Zhang et al., 2022). That is to say, physical activities can not only directly affect the negative emotions of college students, but also indirectly influence negative emotions by enhancing resilience (Zuo et al., 2025). Based on the results of this study, during the process of physical activities, college students can have an indirect impact on reducing the level of depression or preventing the risk of depression by enhancing their resilience levels (such as goal concentration, emotion control, and positive cognition).

Building on this foundation, this study further found that in the mediation model for male students, physical activity has a direct negative relationship with depression levels (β = −0.45), with psychological resilience playing a partial mediating role (β = −0.08). Similarly, in the mediation model for female students, physical activity also exhibits a direct negative effect on depression levels (β = −0.38), and psychological resilience acts as a partial mediator (β = −0.13). In other words, there is a direct or indirect relationship between physical activity and depression levels in both male and female, but previous research findings have shown some discrepancies regarding this perspective. For example, some studies suggest that physical activity levels are only associated with depression in male, but show no significant correlation in female (Goldfield et al., 2011; Xiao et al., 2017). Conversely, other research indicates that regular exercise can alleviate depressive symptoms in patients with mild to moderate depression, and this effect is more pronounced in female than in male (Zhang and Yen, 2015). Based on the findings of this study, we speculate that these discrepancies may be related to specific cultural contexts and sample characteristics. It is worth noting that we found that physical activity exerted greater direct explanatory power on depression in male students, whereas a stronger mediating role was observed through resilience in female students. This suggests that male students may achieve more direct reduction in depression by increasing physical activity levels, while female students' physical activity is more likely to indirectly influence depression levels through the enhancement of psychological resources (such as resilience). Research suggests that males typically regulate physiological states through high-frequency physical activities, thereby effectively alleviating emotional distress—a phenomenon potentially associated with their higher muscle mass and stronger physiological stress responses (Sun Q. F. et al., 2024). In contrast, females can achieve significant emotional improvement even with lower physical activity frequency. This may be attributed to their heightened sensitivity to the mood-regulating effects of physical activity, as well as their tendency to perceive physical activity as a crucial channel for emotional release. This pathway facilitates better stress management and emotional regulation (Liu, 2018), consequently contributing to the reduction of negative emotions such as depression. The results suggest a more pronounced direct linear relationship between physical activity and depression levels in male college students, where increased physical activity volume generally corresponds to lower depression levels. In contrast, the linear association between physical activity and depression in female students appears to be more influenced by psychological factors, demonstrating a greater tendency toward indirect regulation of depressive emotions through psychological resources. For instance, physical activity may enhance emotional regulation capacity indirectly by strengthening resilience levels. Therefore, Hypothesis H4 of this study has been confirmed.

### 4.4 Limitations

This study aims to explore the relationship among physical activity, resilience, and depression levels of college students from the perspective of exercise psychology and reveal the corresponding gender differences. However, as this study adopts a cross-sectional survey design, the findings are primarily based on participants' subjective reports, which may involve potential biases inherent in self-reported data. Subsequent research could incorporate longitudinal empirical studies, utilizing a combination of subjective and objective data to present results, and further enhance scientific rigor through intervention studies. Meanwhile, this study mainly adopted Pearson correlation analysis, general linear regression analysis, and structural equation model to reveal the relationships between variables, but could not effectively reveal the causal relationships among variables, and future research can be enriched by combining more research tools (such as artificial intelligence). Finally, this study mainly examined the mediating role of resilience between physical activity and depression levels of college students of different genders. Future research could explore more mediating or moderating variables (such as academic or environmental factors) to enrich the findings.

## 5 Conclusion

Among college students, male students demonstrate significantly higher levels of physical activity and significantly lower levels of depression compared to their female counterparts. Our study indicates that there are significant correlations among physical activity, resilience, and depression in college students. Specifically, the level of physical activity not only shows a direct negative relationship with depression but also reveals that resilience plays a partial mediating role between the two. Further analysis revealed that the partial mediating role of resilience reached statistical significance in both male and female models. Specifically, physical activity manifest greater direct explanatory power for depression in male students, whereas a stronger mediating relationship was observed through resilience in female students. This difference not only suggests the existence of gender-specific mechanisms in the process through which physical activity alleviates depression, but also implies that the monitoring or evaluation of relevant psychological indicators should be emphasized when designing interventions. Overall, the depression levels of college students are closely associated with various factors (such as physical activity and resilience), and this relationship is susceptible to the influence of gender differences. Therefore, it is advisable to incorporate greater consideration of psychological and gender-related factors in subsequent intervention studies on physical activity. For instance, programs that emphasize direct physical activity for males, and programs that focus on resilience-building aspects (like social interaction, goal setting, and coping skills) through physical activity for females.

## Data Availability

The original contributions presented in the study are included in the article/supplementary material, further inquiries can be directed to the corresponding author.
